# Mice, double deficient in lysosomal serine carboxypeptidases Scpep1 and Cathepsin A develop the hyperproliferative vesicular corneal dystrophy and hypertrophic skin thickenings

**DOI:** 10.1371/journal.pone.0172854

**Published:** 2017-02-24

**Authors:** Xuefang Pan, Yanting Wang, Torben Lübke, Aleksander Hinek, Alexey V. Pshezhetsky

**Affiliations:** 1 Department of Medical Genetics, CHU Sainte-Justine Research Center, University of Montreal, Montreal, Quebec, Canada; 2 Cardiovascular Research Program, the Hospital for Sick Children and Department of Laboratory Medicine and Pathobiology, University of Toronto, Ontario, Canada; 3 Department of Chemistry, Biochemistry I, Bielefeld University, Bielefeld, Germany; 4 Department of Anatomy and Cell Biology, Faculty of Medicine, McGill University, Montreal, Quebec, Canada; Duke University School of Medicine, UNITED STATES

## Abstract

Vasoactive and mitogenic peptide, endothelin-1 (ET-1) plays an important role in physiology of the ocular tissues by regulating the growth of corneal epithelial cells and maintaining the hemodynamics of intraocular fluids. We have previously established that ET-1 can be degraded in vivo by two lysosomal/secreted serine carboxypeptidases, Cathepsin A (CathA) and Serine Carboxypeptidase 1 (Scpep1) and that gene-targeted *CathA*^*S190A*^ */Scpep1*^*-/-*^ mice, deficient in CathA and Scpep1 have a prolonged half-life of circulating ET-1 associated with systemic hypertension. In the current work we report that starting from 6 months of age, ~43% of *CathA*^*S190A*^ */Scpep1*^*-/-*^ mice developed corneal clouding that eventually caused vision impairment. Histological evaluation of these mice demonstrated a selective fibrotic thickening and vacuolization of the corneas, resembling human hyperproliferative vesicular corneal stromal dystrophy and coexisting with a peculiar thickening of the skin epidermis. Moreover, we found that cultured corneal epithelial cells, skin fibroblasts and vascular smooth muscle cells derived from CathA/Scpep1-deficient mice, demonstrated a significantly higher proliferative response to treatment with exogenous ET-1, as compared with cells from wild type mice. We also detected increased activation level of ERK1/2 and AKT kinases involved in cell proliferation in the ET-1-treated cultured cells from CathA/Scpep1 deficient mice. Together, results from our experimental model suggest that; in normal tissues the tandem of serine carboxypeptidases, Scpep1 and CathA likely constitutes an important part of the physiological mechanism responsible for the balanced elimination of heightened levels of ET-1 that otherwise would accumulate in tissues and consequently contribute to development of the hyper-proliferative corneal dystrophy and abnormal skin thickening.

## Introduction

Endothelin-1 (ET-1) is recognized as one of the most potent vasoactive regulators known to date. It modulates blood pressure by inducing constriction of arterial vascular smooth muscle cells (SMCs). ET-1 is also known as a potent mitogen of vascular endothelium and SMCs[1, 2]. While the bulk of ET-1 is secreted by arterial endothelium, it is also produced in central nervous system where its functions remain unclear. In human patients high levels of ET-1 have been associated with systemic and pulmonary hypertension, as well as with diverse cardiovascular disorders [3]. In mice overexpression of human ET-1 resulted in vascular remodelling and endothelial dysfunction[4, 5], whereas ET-1 deficient mice showed respiratory failure at birth and morphological abnormalities of the pharyngeal-arch-derived craniofacial tissues and organs[6].

Significant body of evidence also indicated that fluctuations of ET-1 levels contribute both to the physiology and pathology of eye tissues (reviewed in [7]). The presence of ET-1 has been detected in the human corneal epithelium[8] and implicated in growth regulation of bovine and rabbit corneal epithelial cells [7, 9, 10]. ET-1 is also secreted by the ciliary epithelium and by its tear glands and accumulates in the aqueous humour of the eye bulb, where its level exceeds several times levels detected in plasma[11]. Moreover, it has been documented that injection of exogenous ET-1 causes a dose-dependent vasoconstriction of the retinal arteries[12] and that temporary vasoconstrictions in response to changes in endogenous ET-1 levels contribute to the physiological regulation of retinal hemodynamics (reviewed in[13]). On the other hand, the persisted elevation of ET-1 in the aqueous humour, causing arterial contraction and swelling of the trabecular meshwork, have been linked to building up of the intraocular pressure (glaucoma) in humans as well as in the relevant animal models[14–16]. In addition to these vascular effects, increased levels of ET-1 were implicated in development of permanent pathological changes in the retina, such as initiation of neuronal cell apoptosis, microvascular basement membrane thickening and gliosis (reviewed in[17]). Since the continued elevation of ET-1 could contribute to the above mentioned detrimental ophthalmologic effects, the antagonists of ET receptors, ET-1 neutralizing antibodies or drugs blocking ET-1 synthesis are now considered as potential tools in the pharmacological treatment of glaucoma[18, 19].

Previous studies from our laboratory defined a novel pathway for enzymatic inactivation of circulating ET-1 involving two lysosomal/secreted serine carboxypeptidases, cathepsin A (CathA) and serine carboxypeptidase 1 (Scpep1)[20, 21]. In particular, we showed that mice with double deficiency of CathA and Scpep1 developed a persisted hypertension and demonstrated a prolonged half-life of circulating ET-1 as well as a more pronounced vasoconstriction in response to low pharmacological doses of this peptide[21].

In the current work we provide experimental evidence that physiological inactivation of ET-1 by a tandem of serine carboxypeptidases, CathA and Scpep1 is also prerequisite for maintaining its turnover in the eye cornea and dermis, essential for controlling cell proliferation.

## Materials and methods

### Animals

Mice containing Ser190Ala point mutation in the CathA active site (CathAS190A strain) and those with the Scpep1 gene interrupted by gene-trap technology (Scpep12/2 strain) were generated as previously described[21, 22]. Mice were housed in an enriched environment with continuous access to food and water, under constant temperature and humidity, on a 12 h light/dark cycle. Approval for the animal care and the use in the experiments was granted by the Animal Care and Use Committee of the Ste-Justine Hospital Research Center.

### Histological examination of mouse tissues

Eyes were dissected from adult 6–8 month-old WT and *CathA*^*S190A*^/*Scpep1*^*-/-*^ mice (N = 8 for each group) and then fixed in 4% paraformaldehyde. The transversal histological sections derived from eyes and skin of normal and transgenic mice were stained with hematoxyline and eosine, as well as with the pentachrome Movat’s method that visualizes collagen (yellow) and elastin (black) as previously described[23]. Adjacent sections were stained with monoclonal antibody to chondroitin sulfate (Sigma; 10 μg/ml) and with polyclonal antibody to collagen type I (Chemicon, Billerica; 4 μg/ml) to further test whether the macroscopically observed changes in knockout mice are associated with increased cellular proliferation and/or accumulation of the peculiar extracellular matrix.

### Cell isolation and cultures

Skin fragments and entire length aortas, derived from 5–6 mouse, were minced in a DMEM containing collagenase type I (GIBCO, 17100–017, 3 mg/ml), trypsin (Sigma T-1426, 0.5 mg/ml), and DNAse type I (Sigma D-4263, 20 μg/ml), incubated at 37°C for 2 h. The isolated cells were then centrifuged for 5 min at 1000 g and 4°C, re-suspended in 10 ml of DMEM containing 10%FBS, 1% antibiotic-antimycotic (GIBCO 15240–062), 0.5% Fungizone (GIBCO 15290–018), plated in culture dishes and subsequently maintained under 5% CO_2_ at 37°C. The medium was changed every three days. After 3 passages 100% of aortic smooth muscle cells (ASMC) were positive to the specific marker, smooth muscle α-actin as assayed by FACS with A 2547 antibody (Sigma).

Corneal epithelial cells were isolated and cultured as described by Kobayashi et al. [24]. Eyes from 5 euthanized mice of the same sex, and genotype were enucleated and incubated in DMEM/F12 (1:1 mixture; Thermo Fisher Scientific 11320033) containing 15 mg/ml of dispase II (Sigma 4942078001), 0.2% Fungizone (Gibco 15290–018), and 1% antibiotic-antimycotic (Gibco 15240–062) on the rocking platform for 18 h at 4°C. The loosened corneal epithelial sheets were peeled off with forceps and incubated in 100 μl of 0.25% trypsin (Invitrogen) for 10 min at 37°C. The activity of trypsin was inhibited by adding 100 μl of 2 mg/ml soybean trypsin inhibitor (Roche Diagnostics 10109886001) solution in PBS and the sheets were separated into single cells by pipetting. Then 2 ml of low-calcium, low-bovine pituitary extract (BPE) serum-free progenitor cell targeted medium (CNT-57; CELLnTEC, Bern, Switzerland) was added to the isolated cells. The cells were cultured in type-I collagen-coated 35 mm plastic dishes (Corning 354456) at 37°C under 95% humidity and 5% CO_2_. The medium was changed every 2 to 3 days. The cell purity analyzed by immunocytochemistry with anti-cytokeratin 12 (L-15) antibodies (Santa Cruz Biotechniology Inc, sc-17101) SANTA was >80%.

### Detection of proliferating corneal epithelial cells, fibroblasts and myofibroblasts by immunocytochemistry

After the second passage, corneal epithelial cells and mouse skin fibroblasts were plated in 35 mm culture dishes (10^5^ cells/dish) and maintained in CNT-57 and DMEM/10% FBS medium, respectively. After reaching ~70% of confluency cells were incubated in the media deprived of serum or growth factors in the presence or absence of 2 nM of ET-1. The cells were fixed and permeabilized in 100% methanol at -20°C and then stained either with monoclonal antibody to Proliferating Nuclear Antigen (DACO, Glostrup, Denmark; final concentration 10 μg/ml) or with monoclonal anti-α-actin antibody (1A4: sc-32251, Santa Cruz Biotechnology, Dallas TX; final concentration 10 μg/ml) followed by fluorescein-conjugated goat anti-mouse secondary antibody (Sigma-Aldrich Canada, Oakville ON). Nuclei were counterstained either with propidium iodide or DAPI (both from Sigma-Aldrich). The parallel sets of fibroblast cultures were co-stained with a monoclonal mouse anti-α-actin antibody (final concentration 10 μg/ml) and the polyclonal rabbit anti-vimentin (H-84, sc-5565; final concentration 15 μg/ml) antibodies (both from Santa Cruz Biotechnology) followed by rhodamine-labeled goat anti-mouse and fluoresceine-labeled goat anti-rabbit secondary antibodies. Slides were examined using a Nikon Eclipse E1000 microscope equipped with a cooled CCD camera (QImaging, Retiga EX) and analyzed with Image-Pro Plus software (Media Cybernetics, Silver Springs, MD) to calculate the proportion of antigen-positive cells.

The ratio of the PCNA-positive (proliferating cells) to total number of cells, detected with propidium iodide, as well as numbers of the vimentin-positive fibroblasts and α-actin-positive antibody were calculated and statistically evaluated using the Student’s t-test. The P-value of 0.05 was considered significant.

### Analysis of ERK1/2 and AKT phosphorylation by Western blot

Fibroblasts or ASMC cultured in 100 mm dishes to confluent layer were incubated overnight in a serum-free DMEM, and treated for 5 min with 100 nM ET-1. To test the pharmacological inhibition of the ET-1 receptors, the cells were pre-treated for 30 min with 2 μM BQ610 (EMD 203715) or BQ788 (EMD 5223838) before the stimulation with 100 nM ET-1. To test the pharmacological inhibition of MEK1 activation, the cells were pre-treated for 30 min with 10 μM U0126 (Cell Signalling 9903) or 25 μM PD98059 (Cell Signalling 9900) before the stimulation with 100 nM ET-1. Then cells were washed with ice-cold PBS and lysed in RIPA (RadioImmunoPrecipitation Assay) buffer containing 50 mM Tris HCl, pH 7.4, 150 mM NaCl, 1% NP-40, 0.25% sodium deoxycholate, 0.1% SDS, 2 mM EDTA, 1 mM PMSF, protease and phosphatase inhibitor cocktails (Roche 04693132001 and 04906837001). Cell lysates were analyzed by Western blot using anti-phospho-ERK1/2 (Cell Signalling 4376, dilution 1:1000), anti-phosphoSer473-AKT (Cell Signalling 9271, dilution 1:1000), anti-ERK1/2 (Cell Signalling 4695, dilution 1:1000) or anti-AKT antibodies (Cell Signalling 9272, dilution 1:1000). Detection was performed with anti-rabbit IgG antibodies-HRP conjugate (Cell Signalling 7074S), and the enhanced chemiluminescence reagent (Thermo 32106).

### Measurement of ET-1 induced cell proliferation by MTT assay

The corneal epithelial cells, ASMC or skin fibroblasts were seeded into 96-well plates at a density of 1×10^5^ cells per well and incubated at 37°C for 24 h and then overnight in the medium free of the serum or growth factors. The cells were treated with ET-1 at a concentration of 50, 100, 200 and 400 nM for 24 h in the medium containing 1% FBS. Then the medum was removed, 100 μl of fresh serum-free medium and 10 μl of MTT solution were added to each well followed by incubation at 37°C, under 5% CO_2_ for 3 h to allow the MTT to be metabolized. Medium was further removed and MTT metabolic product, blue formazan, was dissolved in 100 μl of DMSO. After shaking the plate for 5 minutes, optical density was measured at 570 nm using a plate reader. Background optical density at 620 nm was subtracted from the data to normalize for opacity of the samples.

In the experiments with pharmacological inhibitors, cells were cultured in 96-well culture plates to 50% confluence. Starved and then inhibitor-treated or untreated cells were further incubated for 24 h or 48 h in 1% FBS DMEM containing 100 nM ET-1 and different concentrations of the inhibitors (see above). The number of viable cells in each well was estimated by the measurement of the rate of mitochondrial metabolism of MTT using a cell proliferation assay kit.

### ^3^H-Thymidine incorporation assay

DNA synthesis was evaluated by incorporation of [^3^H] thymidine into cells. For [^3^H]-Thy incorporation assays cells were plated in triplicate in 12-well plates. After 24 hours, at approximately 70% confluency, fibroblasts or VSMC were washed twice with PBS and then starved in serum-free DMEM medium overnight. Cells were stimulated with 100 nM ET-1 for 24 hours and during the last four hours in the presence of 1μCi/ml [^3^H]-Thy. Inhibitors were added to the cells prior to the ET-1. To assess [^3^H]-Thy incorporation cells were washed twice with ice-cold PBS, incubated 20 minutes on ice with 10% TCA, and washed with H_2_O. Precipitated DNA was then re-suspended in 0.2 N NaOH and counted in a liquid scintillation counter.

### Measurement of ET-1 level in mouse aqueous humour

Aqueous humour was collected from eyes of five 3–4 month-old mice (25–35 g BW) of same genotype immediately after sacrifice by microscope-guided puncture with a 30-gauge needle and a capillary attraction with 10-μl micropipettes as described[25] and then pooled into a microcentrifuge tube. Quantitative assay of ET-1 was performed using an ELISA kit (Enzo Life Sciences ADI-900-020A) as described by the manufacturer.

### Statistical analysis

Statistical analysis has been performed by one or two-way ANOVA tests using Prism Graphpad software. P-value of 0.05 or less was considered significant. If significance was determined we used Bonferroni post-hoc test to compare specific means

## Results

### Corneal fibrosis and thickening of epidermis in mice with dual CathA/Scpep1-deficiency

*CathA*^*S190A*^ */Scpep1*^*-/-*^ mice with a combined deficiency of CathA and Scpep1 (*CathA*^*S190A*^ */Scpep1*^*-/-*^ mice) were generated by crossing *CathA*^*S190A*^ and *Scpep1*^*-/-*^ mouse strains each in C57BL/6NCrl genetic background [21]. *CathA*^*S190A*^ */Scpep1*^*-/-*^ mice showed a normal growth and behaviour. Both males and females were fertile and could be bred to produce knockout litters. However, starting from 6 months of age *CathA*^*S190A*^ */Scpep1*^*-/-*^ mice showed macroscopically visible corneal clouding in ~43% (6 out of 14) that caused an apparent vision problems. The severity of these problems has been estimated by the visual placing test where the animals, lifted by the tail, where slowly moved horizontally towards the edge of a Plexiglas box[26]. In contrast to WT mice that normally extend their front legs in order to get hold of the box while approaching its edge, the *CathA*^*S190A*^ */Scpep1*^*-/-*^ mice, having visible corneal clouding, extended legs only after their noses or vibrissae physically contacted with the hard edge. Importantly, neither corneal clouding nor physical signs of vision problems were ever detected in WT mice or mice with a single *CathA* or *Scpep1* deficiency (~50 animals analyzed).

**Fig 1:**
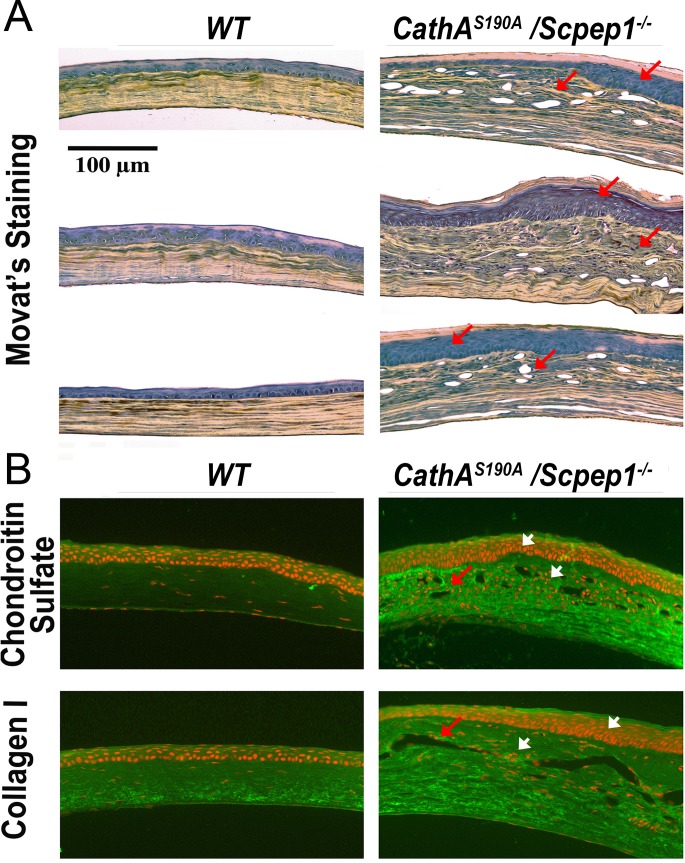
Vesicular corneal dystrophy and ocular fibrosis in mice with combined CathA/Scpep1 deficiency. Eyes were dissected from adult 6–8 month-old WT and *CathA*^*S190A*^/*Scpep1*^*-/-*^ mice with vision problems and fixed in 4% paraformaldehyde. The transversal histological sections derived from each eye were studied by bright field microscopy after pentachrome Movat’s staining that visualized back elastin, yellowish collagen and greenish proteoglycans (**A**) or stained with propidium iodine and antibodies against chondroitin sulfate or collagen Type 1 (**B**) and studied by fluorescent confocal microscopy. Both methods indicated that corneas of *CathA*^*S190A*^/*Scpep1*^*-/-*^ mice showed a selective fibrotic thickening and vacuolization of the corneal stroma (arrows), resembling a vesicular corneal dystrophy in aging humans as well as augmented density of cell nuclei stained with propidium iodide (arrowheads). None of the examined corneas from the age-matched WT mice showed these pathologic features. Panels show representative images of tissues from three different WT and three double deficient mice. Optical magnification 200 x; scale bar equals 100 μm.

The microscopic examination of histological sections stained with the Movat’s pentachrome method revealed pathological changes in the cornea of *CathA*^*S190A*^ */Scpep1*^*-/-*^ mice. In contrast to WT mice, all mice bearing a double CathA/Scpep1 deficiency demonstrated a significant thickening of their corneas, which contained more cells and more extracellular matrix, both in their epithelial and stromal layers, as well as the peculiar vesicular changes, mostly located under the superficial epithelial basement Bowman membrane, and often causing the focal discontinuity in the anterior corneal stroma of *CathA*^*S190A*^ */Scpep1*^*-/-*^ mice (Fig 1A). The higher magnification of H&E stained corneal sections (S1 Fig) further depicted high cellularity of the vacuolated corneal stroma only in double deficient mice. In addition, we detected that 3 of 6 mice from the *CathA*^*S190A*^ */Scpep1*^*-/-*^ group revealed the presence of the extreme epithelial hypertrophy, as well as a thickening of the corneal stroma that jointly contributed to a frontal corneal deformations that resembled the keratomas. Further immunohistochemical analysis of adjacent sections stained with antibodies recognizing several components of extracellular matrix demonstrated that corneas and stromas of the *CathA*^*S190A*^ */Scpep1*^*-/-*^ mice contained a heightened levels of chondroitin sulfate and collagen Type I, as well as augmented density of cell nuclei stained with propidium iodide (Fig 1B). Together, the described findings suggested that morphological changes in the cornea mostly developed in the result of the increased proliferation of stromal fibroblasts that was always associated with the heightened accumulation of the new fibrotic extracellular matrix. At the same time, eyes of *CathA*^*S190A*^ */Scpep1*^*-/-*^ mice did not reveal pathologic changes in their scleras, retinas or lenses, except for scarce focal vacuolization in sclera that was also detected in the WT mice and likely reflected their normal aging (S2 Fig).

**Fig 2:**
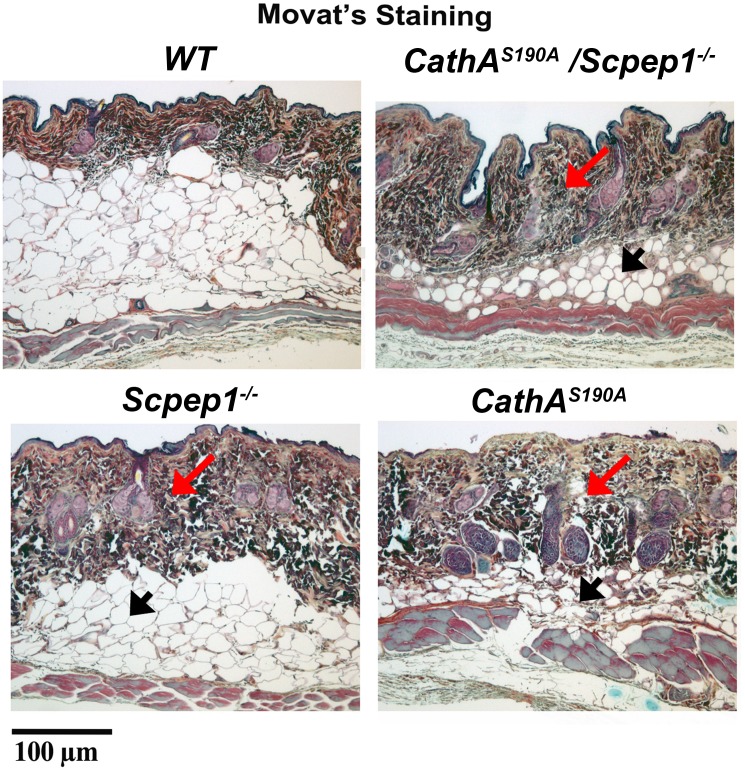
Representative images of transversal sections of abdominal skin fragments derived from WT 6 month-old mouse and age-matched counterparts with indicated selective or combined CathA/Scpep1 deficiencies. Sections are stained with the pentachrome Movat’s method that visualized back elastin, yellowish collagen and greenish proteoglycans. Mice, either with the single or double deficiencies of *CathA*^*S190A*^ *and Scpep1*^*-/-*^ have thicker skin that contains enlarged hyperplastic epidermal glands as well as more extracellular collagen Type I and elastin as compared with their WT counterparts (arrows) suggesting the dermal hypertrophy. Tissues of all tested knockout mice also demonstrate remarkable reduction of dermal fat (arrowheads). Scale bar equals 100 μm.

Importantly, microscopic examination of the skin indicated that 6 month-old mice with either a single or double deficiencies of CathA and Scpep1 had a significantly thicker hypertrophic skin that contained enlarged hyperplastic epidermal glands and more extracellular collagen Type I and elastin (detected with Movat staining) then their WT counterparts (Fig 2). We also observed that dermal hypertrophy was associated with the remarkable reduction of dermal fat tissue, but the relationship of these phenomena is not obvious.

### Corneal epithelial cells, fibroblasts and smooth muscle cells from *CathA*^*S190A*^*/Scpep1*^*-/-*^ mice show significantly increased proliferation in response to ET-1 in culture

In order to further elucidate whether the above described pathological changes in corneas and dermis of our experimental mice could be linked to hyper-proliferation of stromal fibroblasts and keratocytes, we tested the cellular growth rates in primary cultures of both cell types, derived from tissues of *CathA*^*S190A*^ */Scpep1*^*-/-*^ mice as well as from those with single deficiencies of CathA (*CathA*^*S190A*^ strain), or Scpep1 (*Scpep1*^*-/-*^ strain) and WT C57BL/6NCrl. Each experimental group contained cells pooled from 5 mice bearing indicated genotype. As a positive control we used aortic smooth muscle cells, which as we previously have demonstrated depend on the expression of CathA and Scpep1 to inactivate the excess of endogenous or exogenous ET-1[20, 21].

**Fig 3:**
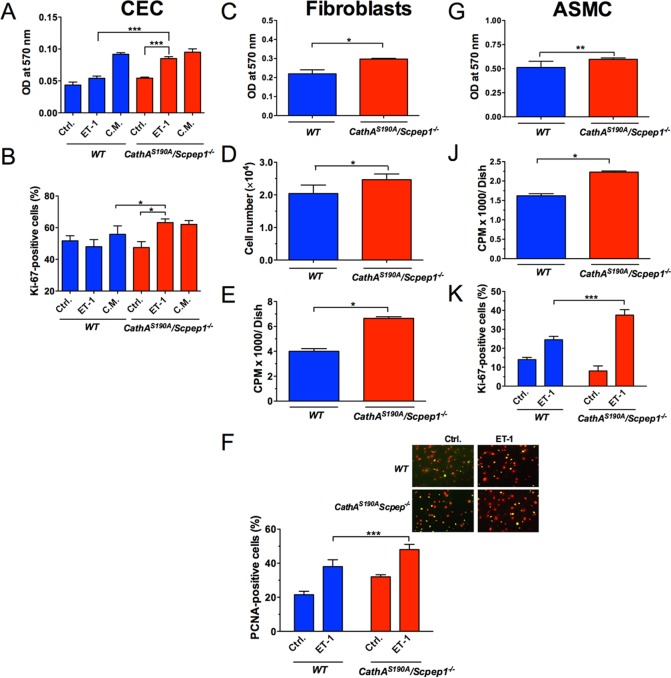
Cultured cells from *CathA*^*S190A*^*/Scpep1*^*-/-*^ mice show significantly increased proliferation response to ET-1. Primary cultures of corneal epithelial cells (CEC) (**A, B**), skin fibroblasts **(C, D, E, F)** and ASMC (**G, J** and **K**) were established by proteolytic digestion of combined corneas, skin tissues and aortas, respectively, extracted from 4 week-old mice of similar sex and genotype. CEC (**A**) and skin fibroblasts (**C, D**) and ASMC (**G**) were seeded into 96-well plates at a density of 1×10^5^ cells per well and incubated at 37°C for 24 h, serum-starved overnight and treated with 100 nM ET-1. After 24 h the concentration of cells was measured by MTT assay (**A**, **C, G**) or cell counting (**D**). The rate of DNA synthesis by fibroblasts (**E**) and ASMC (**J**) in the presence of 100 nM ET-1 was evaluated by incorporation of [^3^H] thymidine. The fraction of cells expressing proliferation markers Ki-67 (**B, K**) and PCNA (**F**) in the serum-free medium **(Ctrl.),** in the presence of 100 nM ET-1 **(ET-1)** or in complete medium (**C.M.**) was analyzed by immunocytochemistry. The panel shows a representative image of skin fibroblasts from *WT* and *CathA*^*S190A*^/*Scpep1*^*-/-*^ mice untreated (**Ctrl.**) or treated with 100 nM ET-1 (**ET-1**) and stained with antibodies against PCNA (green) and propidium iodide (red). Bar graphs show (%) of PCNA or Ki67-positive cells measured using ImageJ software. Values are shown as means (±S.E) of at least 3 independent experiments. Two-way repeated measurements ANOVA was used to test differences between the genotypes: significant differences between the mean values in Bonferroni post-test (* *p*<0.05, ** *p*<0.001, *** *p*<0.0001) are shown.

We have initially established that ET-1 had a significant, positive effect on growth rate of the skin fibroblasts in a concentration dependent manner in the range from 50 nM to 100 nM, whereas the further increase of the peptide concentration to 200 and 400 nM did not cause additional induction of proliferation (S3 Fig). The concentration of 100 nM has been, therefore, chosen for all subsequent experiments where the ET-1-induced proliferation rate of cells was compared between studied mouse strains. Our data showed that the proliferation of ET-1-treated corneal epithelial cells from *CathA*^*S190A*^*/Scpep1*^*-/-*^ mice measured by MTT assay, which estimates concentration of live cells by assaying the activities of mitochondrial dehydrogenases was significantly higher than that of the WT mice (Fig 3A). Same affect was also observed for cultured skin fibroblasts (Figs 3C and 2D) and ASMC (Fig 3G). To confirm these results, we further measured the rate of DNA synthesis by detecting the amount of ^3^H-thymidine incorporated by cells incubated for 8 hours in the presence of 100 nM ET-1. We found that the detected rates of ^3^H-thymidine incorporation were significantly higher in cultures of fibroblasts (Fig 3E) and ASMC (Fig 3J) derived from *CathA*^*S190A*^ */Scpep1*^*-/-*^ mice than in those from WT animals. Finally, ET-1-treated corneal epithelial cells, fibroblasts and ASMC were stained with antibodies detecting either the Proliferating Nuclear Antigen (PCNA) or the Ki-67 (MKI67) protein, markers present only in the cells that entered active phases of the cell cycle (G1, S, G2, and mitosis), but absent from resting cells (G0)[27]. Again, the obtained results indicated that fractions of PCNA-positive fibroblasts and Ki-67-positive corneal epithelial cells and ASMC, quantified with Image-Pro Plus software, were significantly higher in cultured cells derived from *CathA*^*S190A*^ */Scpep1*^*-/-*^ mice as compared with those from the WT mice (Fig 3B, 3F and 3K). Altogether the data indicated the greater proliferative response of cells from *CathA*^*S190A*^ */Scpep1*^*-/-*^ mice to the treatment with the same dose (100 nM) of ET-1.

Importantly, we have also established that treatment with ET-1 did not change the ratio between the vimentin-positive fibroblasts and the α-actin/vimentin-positive myofibroblasts in all tested cultures derived from skin of either *CathA*^*S190A*^ */Scpep1*^*-/-*^ or of WT mice (S4 Fig). Also, when cells were grown in the presence of growth factors or 10% FBS the proliferation rate did not significantly differ between the cells derived from *CathA*^*S190A*^ */Scpep1*^*-/-*^ or WT mice (Fig 3A, S5 Fig), suggesting that the reported effect is specific for the ET-1-induced proliferation.

### Cultured fibroblasts of *CathA*^*S190A*^*/Scpep1*^*-/-*^ mice show increased phosphorylation of ERK1/2 and AKT in response to extracellular ET-1

To further elucidate the mechanism responsible for an increased reactivity of *CathA*^*S190A*^/*Scpep1*^*-/-*^ cells to ET-1, we studied cellular signalling pathways triggered by this factor. Since it was technically infeasible to obtain the amount of mouse corneal cells sufficient for the extensive signalling studies we only tested skin fibroblasts and ASMC that similarly to corneal epithelial cells produce ET-1 and express functional ET-1 A and B receptors coupled to proliferative mechanisms[28, 29].

**Fig 4:**
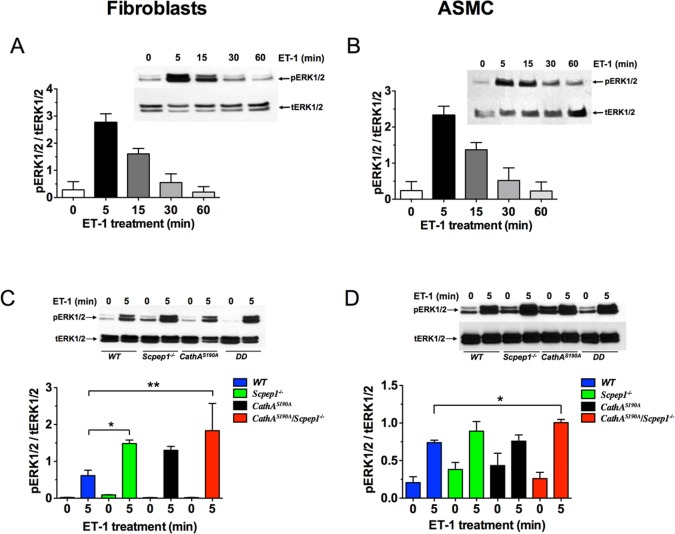
Increased ERK1/2 phosphorylation in ET-1-treated cultured skin fibroblasts and ASMC from *CathA*^*S190A*^*/Scpep1*^*-/-*^ mice. Cultured mouse fibroblasts **(A**) and ASMC **(B)** were serum-starved and treated with 100 mM ET-1 for 0–60 min as indicated on the figure. Total protein extracts were analyzed by Western blotting using antibodies specific to ERK1/2 phosphorylated at Thr202 and Tyr204 residues (pERK1/2) and total ERK1/2 protein (tERK1/2). **(C, D)** Increased ERK1/2 phosphorylation in fibroblasts and ASMC from Scpep1/CathA-deficient mice. Cultured fibroblasts **(C)** and ASMC **(D)** from WT, *Scpep1*^*-/-*^, *CathA*^*S190A*^ and *CathA*^*S190A*^*/Scpep1*^*-/-*^ mice were treated for 5 min with 100 nM ET-1. Total protein extracts were analyzed by Western blotting using antibodies specific for phosphorylated and total ERK1/2 protein. Panels show representative data of 3 independent experiments. Bar graphs show ratios (means and S.D.) of signal intensities for phosphorylated and total ERK1/2 measured using ImageJ software. * *p*<0.05 in paired two-tailed *t*-test.

**Fig 5:**
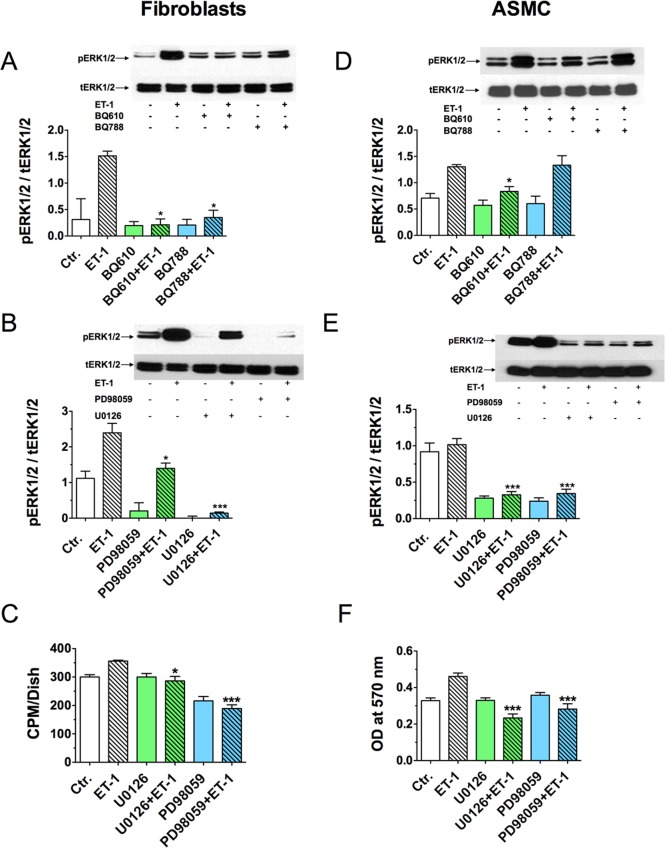
ETA receptor antagonist, BQ610, ETB receptor antagonist, BQ788 and MEK1/2 kinase antagonists, U0126 and PD98059 block both the proliferative effect of 100 nM ET-1 and ERK1/2 phosphorylation. Pharmacological antagonists of ET receptors, BQ610 and BQ788 (**A**) and MEK1/2 kinase antagonists, U0126 and PD98059 (**B**) reduce ERK1/2 phosphorylation in cultured mouse fibroblasts treated with ET-1. In ASMC ERK1/2 phosphorylation was partially reduced by ETR-A antagonist BQ610 (**D**) and significantly inhibited by MEK1/2 kinase antagonists, U0126 and PD98059 (**E**). Serum-starved cultured fibroblasts and ASMC from WT mice were treated or not for 30 min with 2 μM BQ610 and BQ788 or with 10 μM U0126 and 25 μM PD98059 followed by 5 min induction with 100 nM ET-1 as indicated on the figure. Total protein extracts were analyzed by Western blotting using antibodies specific for phosphorylated (pThr^202^/Thr^204^-ERK1/2) and total ERK1/2 protein. Panel shows representative data of 3 independent experiments. Bar graphs show ratios (means and S.D.) of signal intensities for phosphorylated and total ERK1/2 estimated with ImageJ software. * *p*<0.05 in paired two-tailed *t*-test. (**C, F**) Inhibitors of MEK1/2 kinase, U0126 and PD98059 block ET-1 induced proliferation of skin fibroblasts. Cultured fibroblasts from WT mice were serum-starved overnight and treated for 24 h with 100 nM ET-1 in the medium containing 1% FBS in the presence or absence of 2 μM BQ610 or BQ788 as indicated on the figure. Cell proliferation was measured by incorporation of [^3^H] thymidine (**C**) and MTT assay (**F**) as described in the Materials and Methods.

It has been previously described that an interaction of ET-1 with its receptors ETR-A and ETR-B on the cell surface leads to phosphorylation and activation of MEK1/2 and phosphatidylinositol 3-kinase (PI3K), which further phosphorylate and activate extracellular signal-regulated protein kinase 1/2 (ERK1/2) and protein kinase B (AKT), respectively[30–34]. Both further stimulate synthesis of cell growth regulators such as cyclin D1 (reviewed in[35]). We therefore, compared levels of ERK1/2 and AKT phosphorylation in fibroblasts and ASMC before or after the treatment with ET-1 for the *CathA*^*S190A*^/*Scpep1*^*-/-*^ and WT strains of mice. The cells preincubated for 16 h in serum-free medium were treated or not with 100 nM ET-1, harvested and analyzed by Western blot using antibodies against ERK1/2 phosphorylated at Thr202 and Tyr204 residues (pERK1/2) and against total ERK1/2 protein or against AKT phosphorylated on pSer473 (pAKT) and total AKT protein. First, phosphorylation of ERK1/2 was examined at different time intervals after addition of 100 nM ET-1. We found that the level of ERK1/2 phosphorylation in both fibroblasts and ASMC reached a peak at 5 min after exposure of cells to ET-1 (Fig 4A and 4B). Thereafter, the activities of ERK1/2 induced by ET-1 rapidly declined, and returned to baseline control values at 60 min after the stimulation. We therefore have chosen the 5-min time point to compare the ERK1/2 phosphorylation between different cell lines and found that the cells from *CathA*^*S190A*^/*Scpep1*^*-/-*^ mice had significantly higher level of ET-1 induced ERK1/2 phosphorylation than the cells from WT mice (Fig 4C and 4D). Importantly, treatment of the cells with the ET-1 receptor antagonists BQ610 (the antagonist of ET-1 receptor type A, ETR-A) and BQ788 (the antagonist of ET-1 receptor type B, ETR-B) blocked phosphorylation of ERK1/2 in cultured fibroblasts (Fig 5A). ETR-A antagonist, BQ610 also reduced ERK1/2 phosphorylation in ASMC whereas BQ788 did not (Fig 5D). Similar difference in the action of ETR antagonists was previously described in human VSMC by Chen et al. [33]who suggested that ET-1-induced activation of ERK1/2 in these cells is predominantly mediated by ETR-A receptors. In order to further elucidate the link between the increase in the ET-1-mediated phosphorylation of ERK1/2 and the subsequent augmentation of cellular proliferation, we additionally treated cultured cells with PD98059 and U0126, the pharmacological inhibitors of the MEK1/2 kinase, acting upstream of the ERK1/2 in the ET-1 signalling pathway[30–34]. We found that pre-treatment of fibroblasts with PD98059 and/or U0126 reduced both ERK1/2 phosphorylation (Fig 5B) and the cell proliferation, measured with ^3^H-thymidine incorporation (Fig 5C) or MTT assay (Fig 5F). Similar effect was also observed for cultured ASMC (Fig 5E).

**Fig 6:**
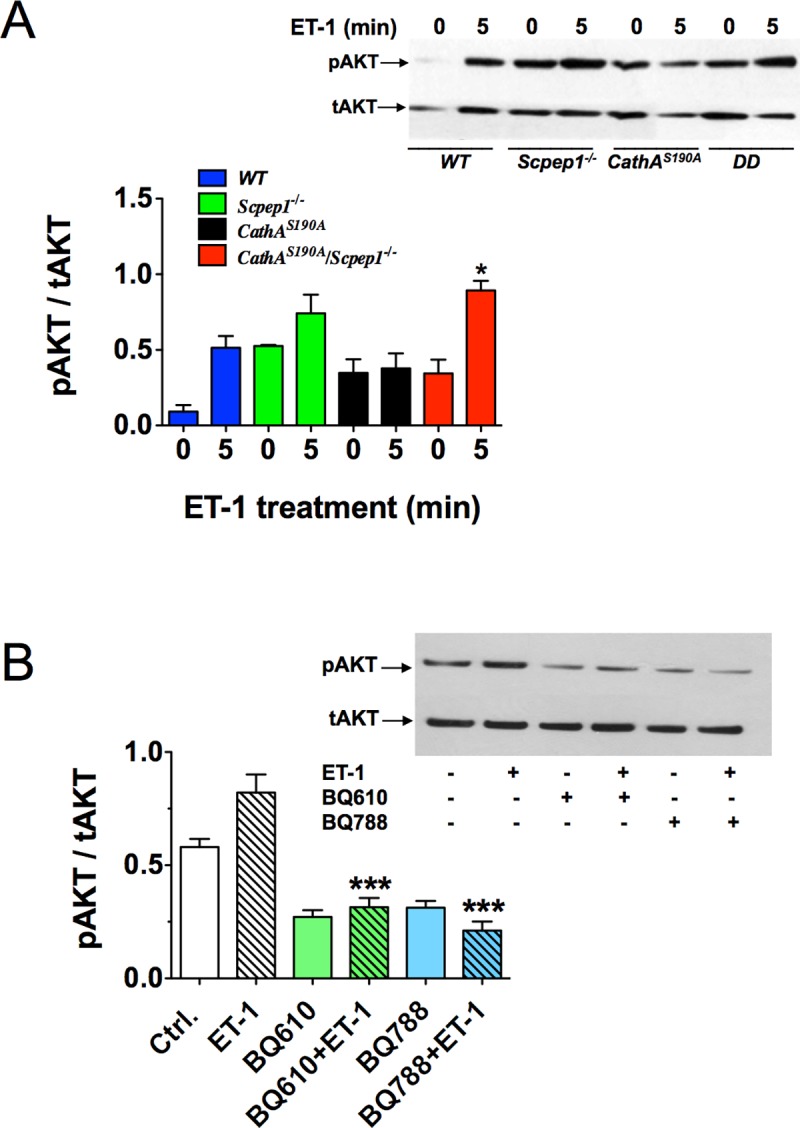
Increased AKT phosphorylation in ET-1-treated cultured skin fibroblasts from CathA^S190A^/Scpep1^-/-^ mice. **(A)** Increased AKT phosphorylation in fibroblasts from CathA/Scpep1-deficient mice. Cultured fibroblasts from WT, *Scpep1*^*-/-*^, *CathA*^*S190A*^ and *CathA*^*S190A*^*/Scpep1*^*-/-*^ mice were treated for 5 min with 100 nM ET-1. Total protein extracts were analyzed by Western blotting using antibodies specific for phosphorylated (pAKT) and total AKT protein (tAKT). **(B)** Pharmacological antagonists of ET receptors, BQ610 and BQ788 reduce AKT phosphorylation in cultured mouse fibroblasts treated with ET-1. Cultured fibroblasts from WT mice were treated or not for 30 min with 2 μM BQ610 or BQ788 followed by 5 min induction with 100 nM ET-1 as indicated on the figure. Total protein extracts were analyzed by Western blotting using antibodies specific for phosphorylated (pAKT) and total AKT protein. Panels show representative data of 3 independent experiments. Graphs below the panels shows ratios (mean values and S.D.) of signal intensities for phosphorylated and total AKT protein measured using Image J software. * *p*<0.05 in paired two-tailed *t*-test.

Treatment with ET-1 also resulted in a significantly higher phosphorylation levels of the AKT kinase in cultured fibroblasts from *CathA*^*S190A*^/*Scpep1*^*-/-*^ mice as compared to those from control WT animals and the effect was even higher than that for ERK1/2 (Fig 6A). AKT together with ERK1/2 is involved in proliferation signaling in fibroblasts and mediates a parallel signaling pathway [33, 36]. Importantly, its phosphorylation was also inhibited by the ETR antagonists BQ610 and BQ788, demonstrating that it is specific for ET-1 action on the cells (Fig 6B).

## Discussion

CathA, one of the most abundantly expressed mammalian carboxypeptidases[37, 38], has a well-characterized allosteric function in the lysosome, where it forms a complex with lysosomal neuraminidase 1 (Neu1) and β-galactosidase 1 (GBA). This complex supports enzymatically active conformation of Neu1 and protects both Neu1 and GBA against degradation by lysosomal endopeptidases (reviewed in[39]). Genetic deficiency of CathA causes galactosialidosis (OMIM #256540), a severe systemic lysosomal storage disease that manifests with skeletal abnormalities and neurological degeneration (reviewed in[40, 41]). Scpep1 is a recently described lysosomal carboxypeptidase with a high amino acid sequence similarity to CathA that is abundant in kidney, lung and heart [22]. This enzyme has been originally identified by Chen et al., as one of retinoid-inducible genes in rat aortic smooth muscle cells[42]^,^[43].

Previously we generated and characterized the mouse with double deficiency of CathA and Scpep1[21]. These studies showed that both enzymes have a common physiological substrate, ET-1. Mice devoid of both CathA and Scpep1 activities showed significantly reduced degradation rate of ET-1 in the blood, aorta and lungs as compared to WT mice, or those with single deficiencies of CathA or Scpep1. When kept on both normal and high-salt diet mice had significantly higher arterial blood pressure. They also showed bigger change in blood pressure in response to systemic injection of ET-1. Mesenteric arteries from double-deficient mice showed higher ex-vivo constriction in response to ET-1 as compared with those from control, CathA-deficient or Scpep1-deficient animals[21].

Our current results suggest that normal activities of both CathA and Scpep1 may also play an important role in the eye, and skin by balancing ET-1 turnover. We have discovered that about 30% of senescent (6 months and older) CathA/Scpep1-deficient mice exhibited corneal cloudiness that finally led to their blindness. We have also established that these symptoms are associated with the development of a significant corneal thickening and formation of the multiple inclusions between layers of the corneal stroma. These alterations were linked to the augmented proliferation of superficial epithelial cells and stromal fibroblasts as well as increased levels of extracellular matrix components (Fig 1). Together, these pathologic changes strongly resemble those occurring in bilateral non-inflammatory human corneal dystrophies. While the vast majority of inherited corneal dystrophies have been linked to specific mutations (reviewed in[44]), clinically similar symptoms have been also described in the patients afflicted by multiple lysosomal storage diseases including different forms of mucopolysaccharidoses, cystinosis, Fabry disease, and mucolipidosis IV[45–47]. Moreover, corneal clouding and corneal keratomas leading to a vision loss have been reported in CathA-deficient patients diagnosed with the late infantile form of galactosialidosis[48]. These symptoms were believed to be caused by the secondary deficiency of Neu1 resulting in storage of sialylated oligosaccharides and glycopeptides, since similar ophthalmologic problems were also observed in the patients affected with a single Neu1 genetic deficiency, sialidosis (OMIM #256550). Importantly, our current data additionally demonstrate, that corneal clouding also occurs in *CathA*^*S190A*^*/Scpep1*^*-/-*^ mice that as we have previously shown have normal levels of Neu1 activity and do not store sialylated glycoconjugates[21]. Recent publication[49] suggested that corneal dystrophy and clouding in different lysosomal diseases could be linked to impaired autophagy known to be a general consequence of lysosomal storage (reviewed in[50]). In turn, this would impair normal turnover of constantly produced corneal glycosaminoglycans and proteoglycans of the extracellular matrix leading to the changes resembling stromal corneal dystrophy. Further studies are required to verify this hypothesis although our data confirm the increased levels of chondroitin sulfate in the affected corneas.

We speculate that the fibrotic corneal thickening associated with vesicular stromal dystrophy in aging *CathA*^*S190A*^*/Scpep1*^*-/-*^ mice likely develops in the result of prolonged proliferative action of ET-1. ET-1 levels measured in the aqueous humour of *CathA*^*S190A*^*/Scpep1*^*-/-*^ mice showed a trend for increase as compared with WT mice (S6 Fig) but the difference was not statistically significant, consistent with results previously obtained for the blood plasma. However the levels of ET-1 measured in lungs, heart tissues and blood of *CathA*^*S190A*^*/Scpep1*^*-/-*^ mice 15 min after systemic injection of this hormone were significantly higher than those in the WT mice. These data indicated that the ET-1 degradation rate was slower in CathA/Scpep1-deficient mice although there was no sustained elevated level of this hormone.

ET-1 excess is known to play a major role in development of myocardial fibrosis described in several pathological conditions, including hypertension and diabetes[51, 52] (reviewed in[53]). In vitro, fibrosis has been triggered by up-regulation of ET-1 expression in senescent fibroblasts or by their treatment with the exogenous ET-1 peptide[54, 55]. To test if the impaired ET-1 degradation in the cells from CathA/Scpep1-deficient mice could have a similar effect we studied the proliferation response of serum-starved cultured skin fibroblasts and ASMC to exogenous ET-1 and found that in the CathA/Scpep1-deficient cells it was significantly higher than that in WT cells. At the same time the proliferation rates of WT and CathA/Scpep1-deficient cells in the presence of 10% serum were similar. Consistent with these findings, in CathA/Scpep1-deficient cells ET-1 caused significantly increased phosphorylation and activation of ERK1/2 and AKT kinases known to be essential events in the induction of cellular proliferative response.

Our data seem to contradict previously published report by Chen et al.[43], which described that the Scpep1-null mice generated by replacing exons 1 and 2 of the Scpep1 gene with *Neo* cassette show a decrease in medial and intimal cell proliferation. The same study also reported a dramatic decrease in serum-stimulated growth in a murine ASMC line with a ~50% knockdown of the endogenous *Scpep1*. In contrast we found that primary ASMC derived from mice with double Scpep-1/CathA deficiency showed the growth rates similar to those of the WT cells, when maintained in the presence of serum. Further studies are required to verify if this discrepancy is related to a difference in the genetic background of the mouse models.

Importantly, similarly to skin fibroblasts and ASVMC, corneal epithelial cells produce ET-1 and express functional ET-1 A and B receptors coupled to proliferative mechanisms[56, 57]. Moreover, during corneal neovascularization, ET-1 protein and mRNA expression is upregulated in epithelial cells pointing to an involvement of ET-1 and its receptors in corneal angiogenesis[58]. Similarly, both CathA and Scpep1 show high levels of expression in eye tissues including cornea (http://biogps.org/#goto=genereport&id=74617; http://biogps.org/#goto=genereport&id=19025). It has been also shown that CathA activity in human aqueous humour is increased during cataract, diabetes and glaucoma[59]. Since all above pathologies are characterized by induced ET-1 production it is tempting to speculate that CathA is increased to eliminate the excess of this hormone.

In conclusion, our current findings show that serine carboxypeptidases Scpep1 and CathA may constitute an important part of the multi-faceted mechanism controlling development and functioning of the eye and suggest that these two genes may represent additional targets for therapy of hyperproliferative vesicular corneal dystrophies while specific inhibitors of serine carboxypeptidases might enhance healing of corneal epithelial wounds.

## Supporting information

S1 Fig Histological evaluations of corneas from *CathA*^*S190A*^*/Scpep1*^*-/-*^ mice indicate heightened proliferation of epithelium and stromal fibroblasts. Representative pictures of cornea sections stained with H & E method and captured under high optical magnification (400 x). In contrast to corneas from WT mice containing elongated fibroblasts regularly placed between the parallel stromal layers, the corneas of *CathA*^*S190A*^*/Scpep1*^*-/-*^ mice demonstrate significantly higher cellularity in both epithelial and stromal layer that likely reflect the heightened proliferation of both stromal fibroblasts and epithelium. Scale bar equals 50 μm.
(PDF)

S2 Fig Deficiencies of *CathA*^*S190A*^*/Scpep1*^*-/-*^ contribute to selective corneal pathology. Representative images of eye sections from WT and *CathA*^*S190A*^*/Scpep1*^*-/-*^ mice stained with Movat’s method. Apart from the obvious vesicular corneal dystrophy (A) and keratoconus developing on top of the dystrophic cornea (B) the lenses, retinas and scleras from the *CathA*^*S190A*^*/Scpep1*^*-/-*^ mice do not demonstrate any obvious pathology and do not show morphological differences with their age-matched WT counterparts. Scale bar equals 100 μm.
(PDF)

S3 Fig ET-1 induces proliferation rate of ASMVC. ASMV derived from pooled aortic tissues of 6 WT mice were cultured in DMEM containing 10% FBS. After 3 passages the cells were seeded into 96-well plates at a density of 1×10^5^ cells per well and incubated at 37°C for 24 h; then the media was changed to free serum for overnight incubation. The cells were treated with ET-1 at a concentration of 50, 100, 200 and 400 nM for 24 h in 1% FBS media. Then the concentration of live cells was measured using MTT assay as described in Materials and methods. ET-1 shows a significant, positive effect on growth rate of AVSMC in a concentration dependent manner from 50 nM to 100 nM; the further increase of the peptide concentration to 200 and 400 nM does not cause additional induction of proliferation.
(PDF)

S4 Fig Immunodetection of vimentin and α-actin in cultured dermal mouse fibroblasts. The amount of α-actin-positive myoblasts was similar in the cultures derived from skin of WT and *CathA*^*S190A*^ */Scpep1*^*-/-*^ mice and did not depend on the ET-1 treatment.
(PDF)

S5 Fig FBS-induced proliferation rate of ASMVC and dermal fibroblasts. Fibroblasts (A,B) and ASMV (C) were derived from pooled skin and aortic tissues of WT and *CathA*^*S190A*^ */Scpep1*^*-/-*^ mice and cultured in DMEM containing 10% FBS. After 3 passages the cells were seeded into 96-well plates at a density of 1×10^5^ cells per well and incubated at 37°C for 24, 48, 72 and 96 h. Then the amount cells was measured using flow cytometry (A) or MTT assay (B, D).
(PDF)

S6 Fig ET-1 level in mouse aqueous humour. Aqueous humour was collected from eyes of five 3–4 month-old mice (25–35 g BW) of the same genotype immediately after sacrifice by microscope-guided puncture with a 30-gauge needle and a capillary attraction with 10-μl micropipettes and then pooled into a microcentrifuge tube. Quantitative assay of ET-1 was performed using an ELISA kit (Enzo Life Sciences ADI-900-020A) as described by the manufacturer.
(PDF)
