# Supplementary material for: Mice, double deficient in lysosomal serine carboxypeptidases Scpep1 and Cathepsin A develop the hyperproliferative vesicular corneal dystrophy and hypertrophic skin thickenings
Source: PLoS One. 2017 Feb 24;12(2):e0172854. doi: 10.1371/journal.pone.0172854 (PMC5325571; doi:10.1371/journal.pone.0172854)
Supplement: S1 Fig — Representative pictures of cornea sections stained with H & E method and captured under high optical magnification (400 x). In contrast to corneas from WT mice containing elongated fibroblasts regularly placed between the parallel stromal layers, the corneas of CathAS190A/Scpep1-/- mice demonstrate significantly higher cellularity in both epithelial and stromal layer that likely reflect the heightened proliferation of both stromal fibroblasts and epithelium. Scale bar equals 50 μm. (PDF) [file pone.0172854.s001.pdf]

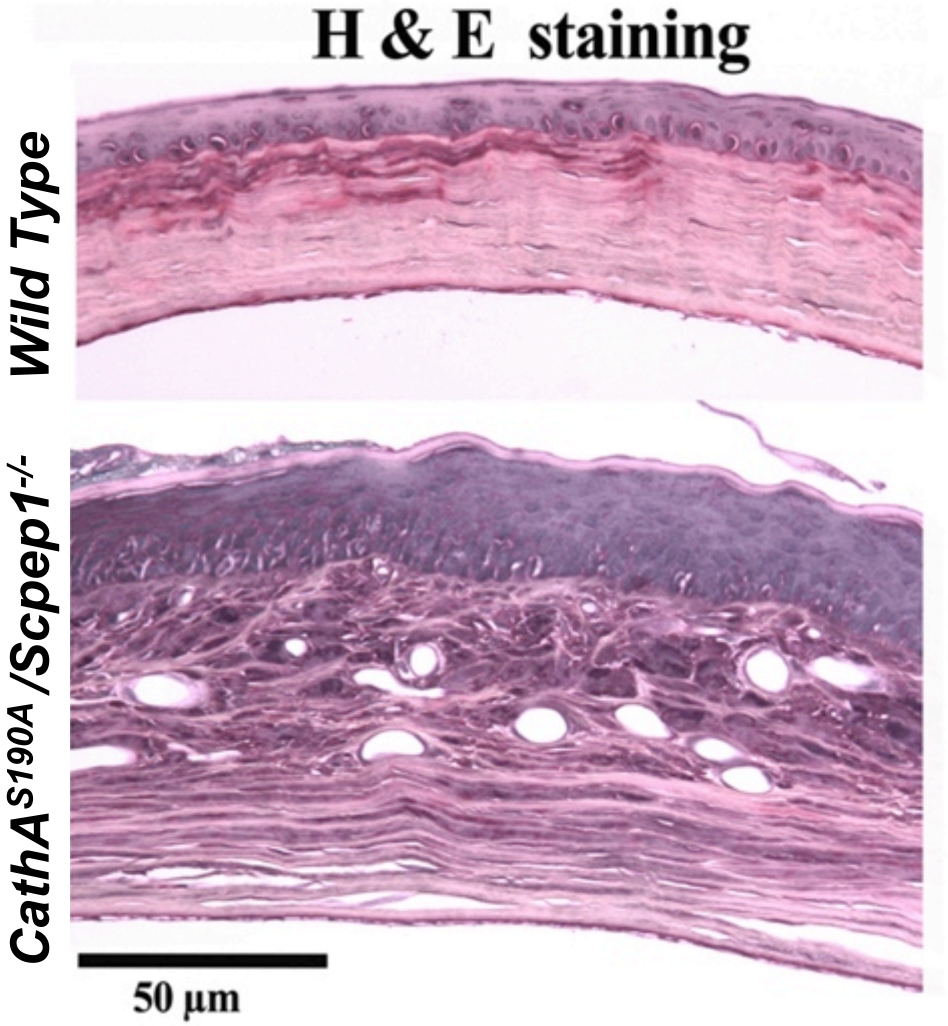

S1 Fig Histological evaluations of corneas from *CathA*^{S190A}*/Scpep1*$^{-/-}$ mice indicate heightened proliferation of epithelium and stromal fibroblasts.

Representative pictures of cornea sections stained with H & E method and captured under high optical magnification (400 x). In contrast to corneas from WT mice containing elongated fibroblasts regularly placed between the parallel stromal layers, the corneas of *CathA*^{S190A}*/Scpep1*$^{-/-}$ mice demonstrate significantly higher cellularity in both epithelial and stromal layer that likely reflect the heightened proliferation of both stromal fibroblasts and epithelium. Scale bar equals 50 µm.
