# Supplementary material for: Mice, double deficient in lysosomal serine carboxypeptidases Scpep1 and Cathepsin A develop the hyperproliferative vesicular corneal dystrophy and hypertrophic skin thickenings
Source: PLoS One. 2017 Feb 24;12(2):e0172854. doi: 10.1371/journal.pone.0172854 (PMC5325571; doi:10.1371/journal.pone.0172854)
Supplement: S2 Fig — Representative images of eye sections from WT and CathAS190A/Scpep1-/- mice stained with Movat’s method. Apart from the obvious vesicular corneal dystrophy (A) and keratoconus developing on top of the dystrophic cornea (B) the lenses, retinas and scleras from the CathAS190A/Scpep1-/- mice do not demonstrate any obvious pathology and do not show morphological differences with their age-matched WT counterparts. Scale bar equals 100 μm. (PDF) [file pone.0172854.s002.pdf]

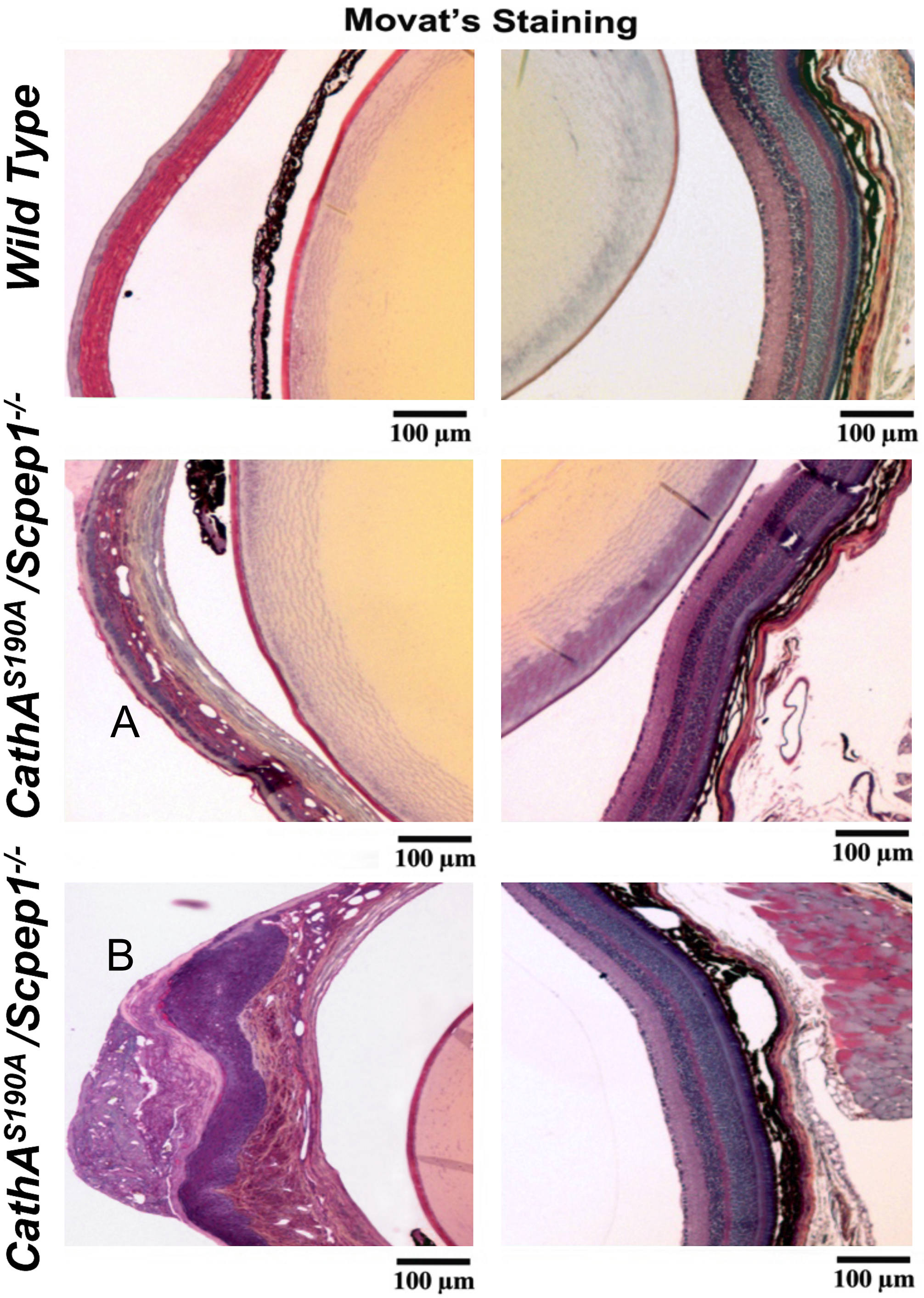


**S2 Fig Deficiencies of *CathA^S190A/Scpep1^-/-* contribute to selective corneal pathology .**

Representative images of eye sections from WT and *CathA^S190A/Scpep1^-/-* mice stained with Movat's method. Apart from the obvious vesicular corneal dystrophy (A) and keratoconus developing on top of the dystrophic cornea (B) the lenses, retinas and scleras from the *CathA^S190A/Scpep1^-/-* mice do not demonstrate any obvious pathology and do not show morphological differences with their age-matched WT counterparts.
Scale bar equals 100 μm
