# Supplementary material for: Mice, double deficient in lysosomal serine carboxypeptidases Scpep1 and Cathepsin A develop the hyperproliferative vesicular corneal dystrophy and hypertrophic skin thickenings
Source: PLoS One. 2017 Feb 24;12(2):e0172854. doi: 10.1371/journal.pone.0172854 (PMC5325571; doi:10.1371/journal.pone.0172854)
Supplement: S3 Fig — ASMV derived from pooled aortic tissues of 6 WT mice were cultured in DMEM containing 10% FBS. After 3 passages the cells were seeded into 96-well plates at a density of 1×10^5^ cells per well and incubated at 37°C for 24 h; then the media was changed to free serum for overnight incubation. The cells were treated with ET-1 at a concentration of 50, 100, 200 and 400 nM for 24 h in 1% FBS media. Then the concentration of live cells was measured using MTT assay as described in Materials and methods. ET-1 shows a significant, positive effect on growth rate of AVSMC in a concentration dependent manner from 50 nM to 100 nM; the further increase of the peptide concentration to 200 and 400 nM does not cause additional induction of proliferation. (PDF) [file pone.0172854.s003.pdf]

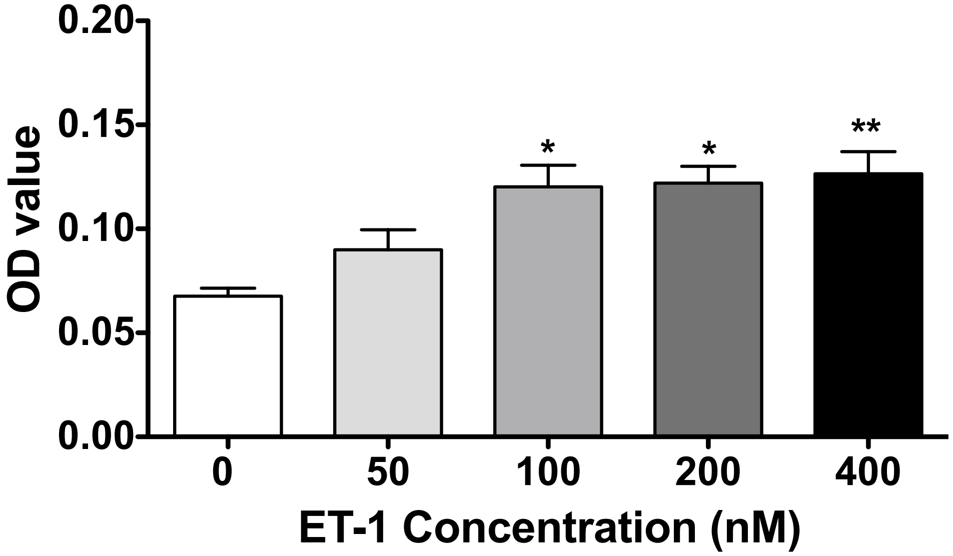

S3 Fig ET-1 induces proliferation rate of ASMVC

ASMV derived from pooled aortic tissues of 6 WT mice were cultured in DMEM containing 10% FBS. After 3 passages the cells were seeded into 96-well plates at a density of 1×10^5 cells per well and incubated at 37 ºC for 24 h; then the media was changed to free serum for overnight incubation. The cells were treated with ET-1 at a concentration of 50, 100, 200 and 400 nM for 24 h in 1% FBS media. Then the concentration of live cells was measured using MTT assay as described in Materials and methods. ET-1 shows a significant, positive effect on growth rate of AVSMC in a concentration dependent manner from 50 nM to 100 nM; the further increase of the peptide concentration to 200 and 400 nM does not cause additional induction of proliferation.
