# Supplementary material for: Mice, double deficient in lysosomal serine carboxypeptidases Scpep1 and Cathepsin A develop the hyperproliferative vesicular corneal dystrophy and hypertrophic skin thickenings
Source: PLoS One. 2017 Feb 24;12(2):e0172854. doi: 10.1371/journal.pone.0172854 (PMC5325571; doi:10.1371/journal.pone.0172854)
Supplement: S4 Fig — The amount of α-actin-positive myoblasts was similar in the cultures derived from skin of WT and CathAS190A /Scpep1-/- mice and did not depend on the ET-1 treatment. (PDF) [file pone.0172854.s004.pdf]

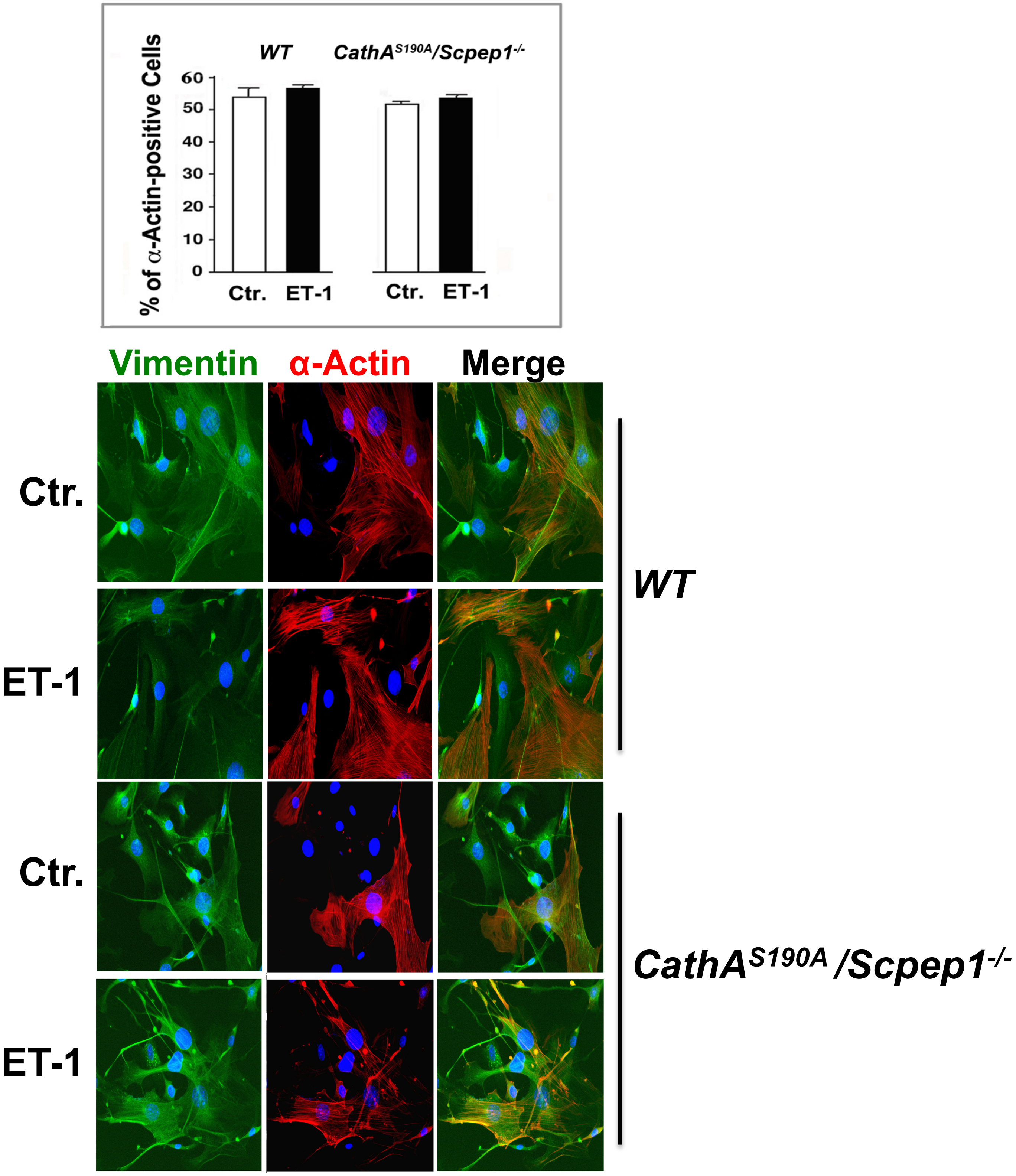

S4 Fig Immunodetection of vimentin and α-actin in cultured dermal mouse fibroblasts

The amount of α-actin-positive myoblasts was similar in the cultures derived from skin of WT and *CathA*^{S190A}*/Scpep1*$^{-/-}$ mice and did not depend on the ET-1 treatment.
