# Supplementary material for: Mice, double deficient in lysosomal serine carboxypeptidases Scpep1 and Cathepsin A develop the hyperproliferative vesicular corneal dystrophy and hypertrophic skin thickenings
Source: PLoS One. 2017 Feb 24;12(2):e0172854. doi: 10.1371/journal.pone.0172854 (PMC5325571; doi:10.1371/journal.pone.0172854)
Supplement: S5 Fig — Fibroblasts (A,B) and ASMV (C) were derived from pooled skin and aortic tissues of WT and CathAS190A /Scpep1-/- mice and cultured in DMEM containing 10% FBS. After 3 passages the cells were seeded into 96-well plates at a density of 1×10^5^ cells per well and incubated at 37°C for 24, 48, 72 and 96 h. Then the amount cells was measured using flow cytometry (A) or MTT assay (B, D). (PDF) [file pone.0172854.s005.pdf]

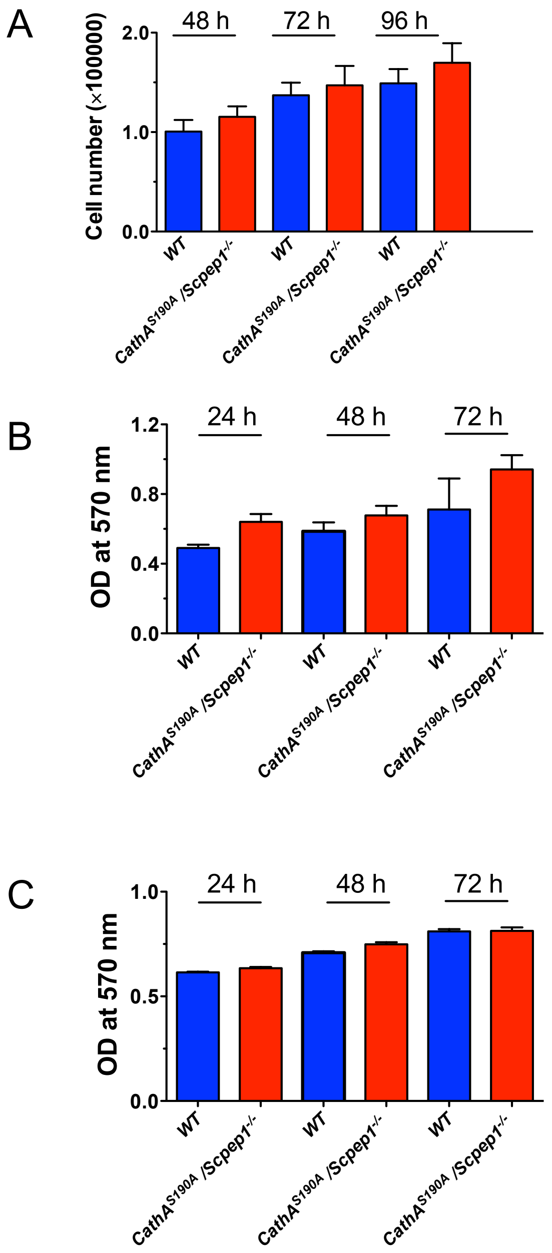

S5 Fig FBS-induced proliferation rate of ASMVC and dermal fibroblasts

Fibroblasts (A,B) and ASMV (C) were derived from pooled skin and aortic tissues of WT and *CathA*^{S190A} */Scpep1*$^{-/-}$ mice and cultured in DMEM containing 10% FBS. After 3 passages the cells were seeded into 96-well plates at a density of 1×10^5 cells per well and incubated at 37 ºC for 24, 48, 72 and 96 h. Then the amount cells was measured using flow cytometry (A) or MTT assay (B, D).
