# Supplementary material for: Mice, double deficient in lysosomal serine carboxypeptidases Scpep1 and Cathepsin A develop the hyperproliferative vesicular corneal dystrophy and hypertrophic skin thickenings
Source: PLoS One. 2017 Feb 24;12(2):e0172854. doi: 10.1371/journal.pone.0172854 (PMC5325571; doi:10.1371/journal.pone.0172854)
Supplement: S6 Fig — Aqueous humour was collected from eyes of five 3–4 month-old mice (25–35 g BW) of the same genotype immediately after sacrifice by microscope-guided puncture with a 30-gauge needle and a capillary attraction with 10-μl micropipettes and then pooled into a microcentrifuge tube. Quantitative assay of ET-1 was performed using an ELISA kit (Enzo Life Sciences ADI-900-020A) as described by the manufacturer. (PDF) [file pone.0172854.s006.pdf]

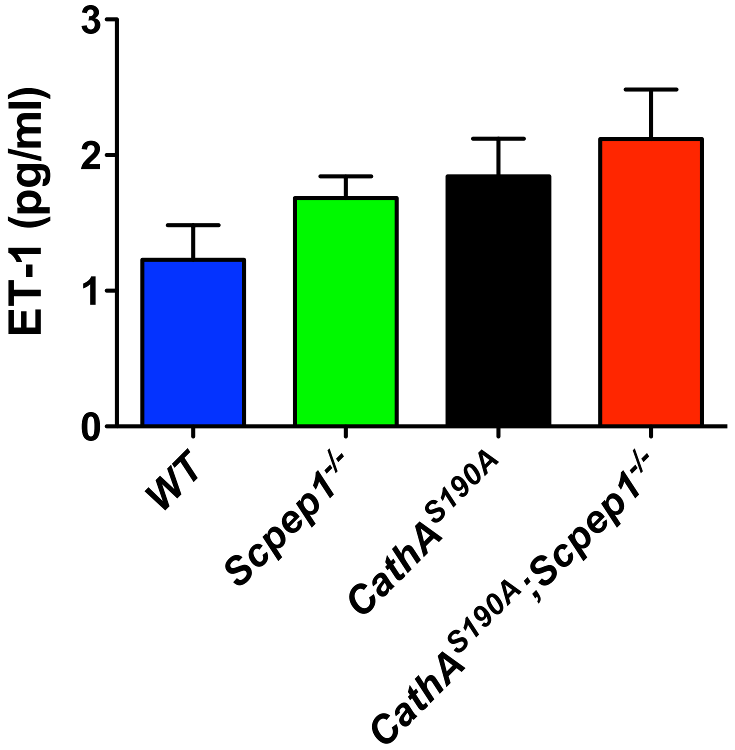

S6 Fig ET-1 level in mouse aqueous humour

Aqueous humour was collected from eyes of five 3-4 month-old mice (25-35 g BW) of the same genotype immediately after sacrifice by microscope-guided puncture with a 30-gauge needle and a capillary attraction with 10-µl micropipettes and then pooled into a microcentrifuge tube. Quantitative assay of ET-1 was performed using an ELISA kit (Enzo Life Sciences ADI-900-020A) as described by the manufacturer.
